# Supplementary material for: Junction Piezotronic Transistor Arrays Based on Patterned ZnO Nanowires for High-Resolution Tactile and Photo Mapping
Source: Sensors (Basel). 2024 Jul 23;24(15):4775. doi: 10.3390/s24154775 (PMC11314929; doi:10.3390/s24154775)
Supplement: Supplementary file 1 [file sensors-24-04775-s001.zip › sensors-3096830-supplementary.pdf]

Supplementary Information

Contents

A. The preparation process for JPT array devices.

B. Single JPT device preparation.

C. Build a test platform.

D. Sapphire with a raised word die aligned with JPT array devices.

E. The photosynaptic properties of JPT structures.

F. SOI back gate-modulated properties.

G. Growth of ZnO nanowires by hydrothermal methods.

A. The Preparation Process for JPT Array Devices

A 25 × 25mm area was cut from a complete SOI wafer as the substrate and then ultrasonically cleaned with anhydrous ethanol, acetone, and deionized water in-turn, followed by compressed air blasting off most of the adhering water, and then dried in a constant temperature chamber at 85°C. In the first step, magnetic sputtering is used to deposit photolithographic markers made from Ni/Au metal on the left and right edges of the substrate. Secondly, the photoresist provides a protective layer, followed by ICP to etch p-type silicon with a length and width distribution of 20 × 80 μm, a pitch of 130 μm, and an array size of 18 × 18 on the substrate (Figure S1a). In the third step, Ni/Au metal is deposited as the source electrode on the right side of the channel by magnetron sputtering, where the electrode covers a 20 × 20 μm area on the right side of the channel (Figure S1b). Fourth, a 60 nm thick 60 × 60 m area Al_2O_3 layer is deposited at the source-drain crossover by using ALD as the insulating layer. Fifth, similar to step three, a layer of Ni/Au is deposited on the left side of the channel as a drain electrode, covering a 20 × 20 m area on the left side (Figure S1d). A p-type silicon channel with a size of 18 × 18 array size and a length and width of 40 × 20 millimeters was prepared as a result of the preceding steps. Sixth, magnetron sputtering was used to deposit polycrystalline ZnO as a seed layer in the channel and to grow ZnO nanowires by hydrothermal means (Figure S1e). Seventh, the sensor's functional area is encapsulated using SU8 photoresist.

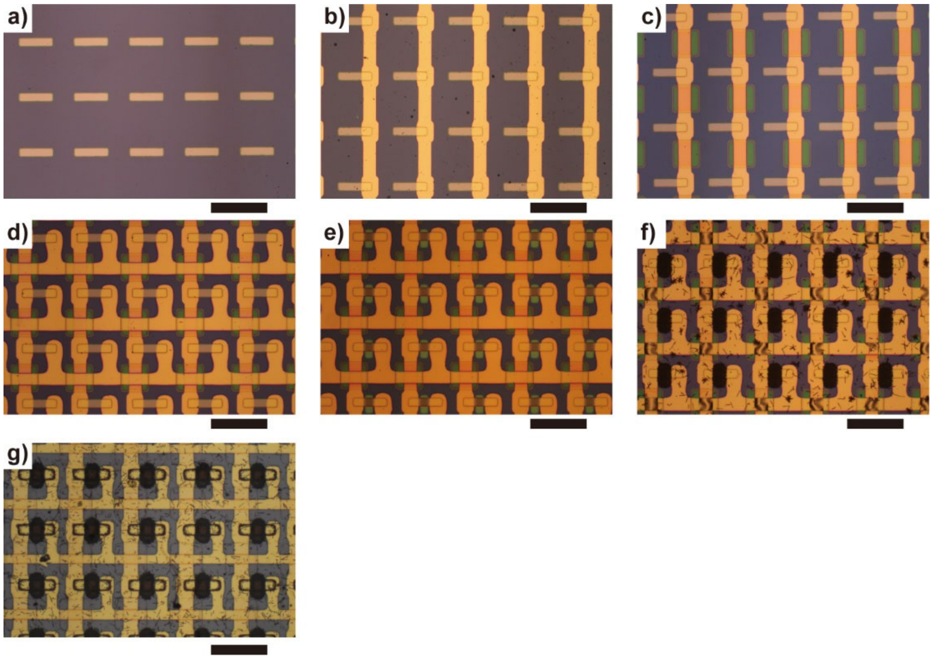

Figure S1. Photograph of the preparation process for the JPT array. ICP etched silicon channel array (**a**). The source electrode was prepared by magnetron sputtering of Ni/Au metal (**b**). ALD is used to grow Al_2O_3 as an insulating layer at the source-drain crossover (**c**). Drain electrode is made of sputtered Ni/Au metal (**d**). Polycrystalline ZnO is sputtered at the center of the channel as the seed layer (**e**). ZnO nanowires hydrothermally grown (**f**) and encapsulated with SU8 (**g**). The scale bar is 130 μm.

B. Single JPT Device Preparation

First, cut off a 25 by 25 mm piece of SOI, and then, clean it with anhydrous ethanol, acetone, and deionized water. Use compressed air to blow off most of the deionized water on the surface of the substrate, and dry it at 85 degrees Celsius. Secondly, lithographic markers consisting of Ni/Au metal are prepared on the prepared substrate using photolithography and magnetron sputtering (Figure S2a). In the third step, a piece of 20 × 80 μm p-Si is etched as the channel material using ICP after applying a photoresist protective layer (Figure S2b,g). Fourth, channel lengths and widths of 40 × 20 μm are obtained after preparing Ni/Au metal electrodes as the source-drain electrodes employing photolithography and magnetron sputtering, respectively (Figure S2c,h). Fifth, a 20 × 40 μm polycrystalline ZnO film is prepared at the channel center as a seed. Sixth, ZnO nanowires are grown vertically at the seed layer location using peristaltic pump-assisted hydrothermal methods. Lastly, use the SU8 photoresist package.

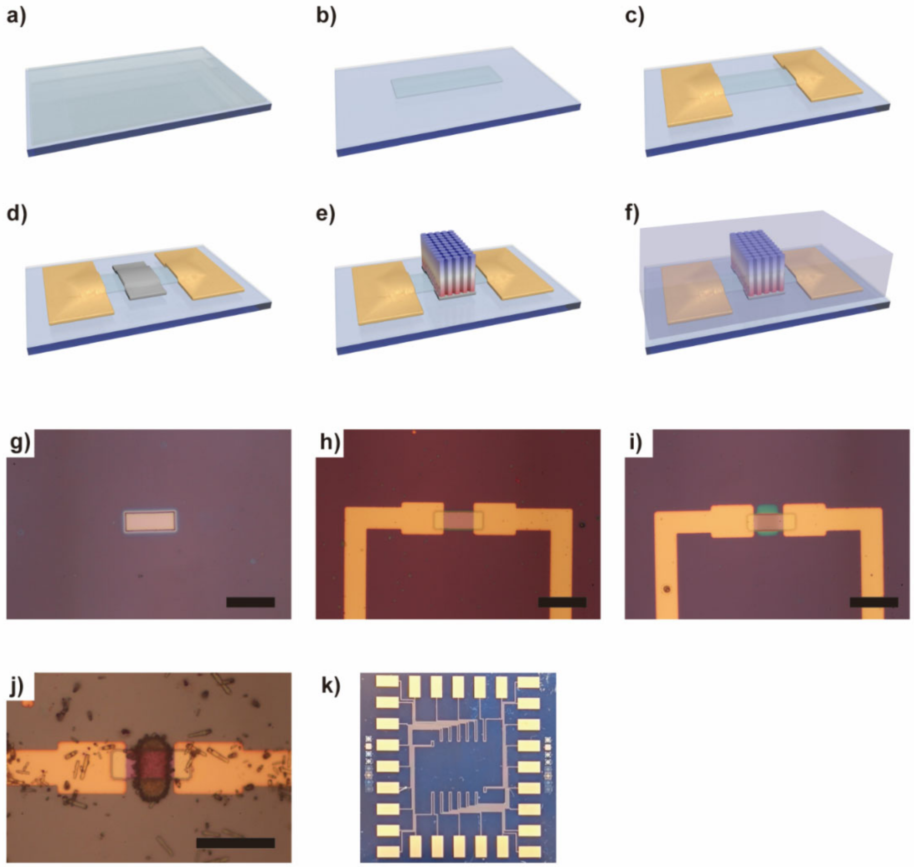

Figure S2. Preparation flow and optical photos of a single JPT device. The preparation flow for the devices (**a**–**f**). The cleaned SOI substrate (**a**). Silicon channels are etched out using ICP (**b**,**g**). Source and drain electrodes (**c**,**h**) are made of sputtered Ni/Au metal. As the seed layer (**d**,**i**), polycrystalline ZnO is sputtered at the center of the channel. Hydrothermal growth of ZnO nanowires (**e**,**j**). The SU8 package (**f**). Device final product (**k**). The scale bar is 80 μm.

C. Build a Test Platform

This test platform consists of control software, a multichannel acquisition system, and a piezo nano-positioning stage. The control software acquires and records electrical signals through a multichannel acquisition system. In mapping the JPT array device to pressure, a piezo nano-positioning stage applies pressure to the device while an electronic scale located below the device records its magnitude (Figure S3a). Flexible PCB and zebra paper are used to connect the prepared devices to the multichannel acquisition system as shown in figure S3b. When testing for light mapping, it is only necessary to replace the lever with an LED light source and power the LED using a constant voltage source.

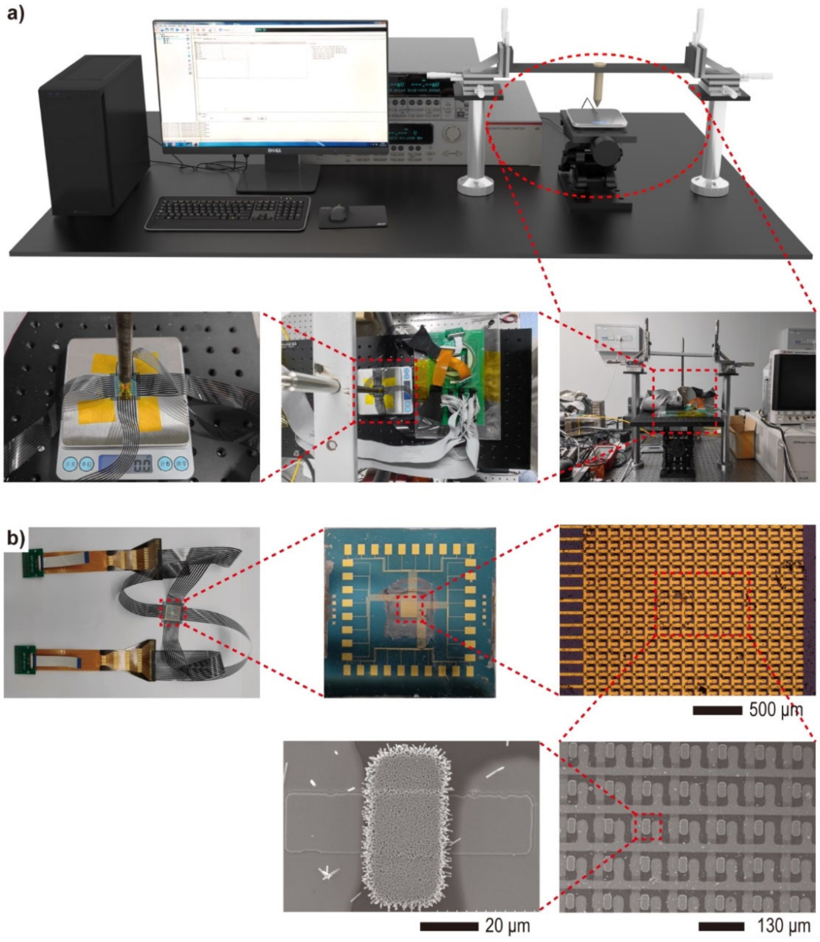

Figure S3. Test platform for the JPT array (**a**) and how the device is connected to the test instrument (**b**).

D. Sapphire with a Raised Word Die Aligned with JPT Array Devices

Under an optical microscope, the sapphire with the "30" bump pattern is aligned with the JPT array device and attached with adhesive tape.

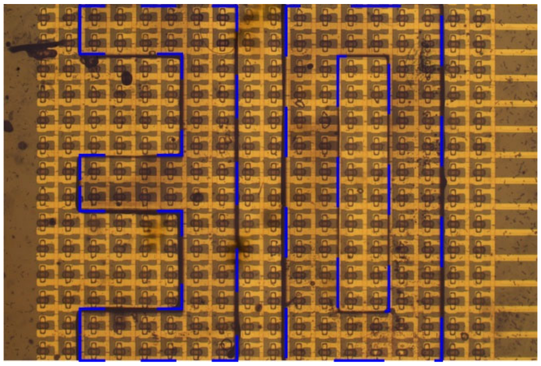

Figure S4. A photograph of the sapphire with the "30" bump pattern after alignment with the JPT array.

E. The Photosynaptic Properties of JPT Structures

Detection of short-wavelength light by JPT structured devices: As a result of ZnO's continuous photoconductivity effect, the carriers generated by photoexcitation in ZnO nanowires may persist for a considerable time. In response to pulsed light at 365 nm wavelength, the JPT structured device showed photosynaptic properties after being stimulated (10 S period, 5 S light duration, light intensity 8.33 mW/cm2). Therefore, the device maintains high conductivity for longer under indoor lighting conditions. This also explains why JPT array devices show different initial currents during stress and optoelectronic testing.

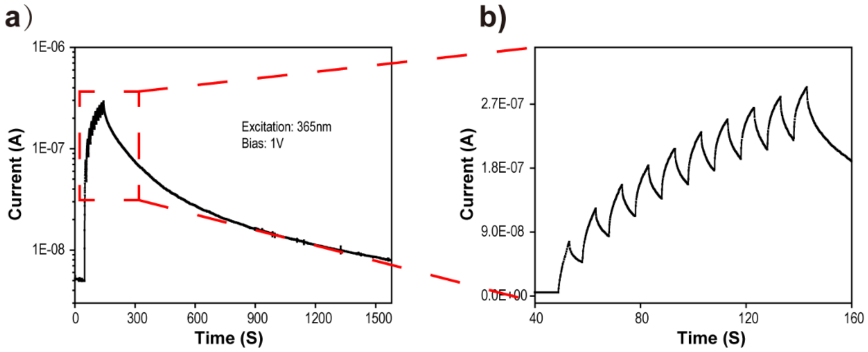

Figure S5. The response of a single JPT device to pulsed light at a fixed bias voltage of 1 V.

F. SOI Back Gate-Modulated Properties

The device was tested for field modulation after the source-drain electrode had been made during the preparation of the single JPT device (Figure S6). Figure S6a,b depict the output curve and transfer curve, respectively. The current between the source and drain electrodes gradually decreases as the back gate voltage changes from negative to positive, demonstrating the channel's hole conduction characteristic.

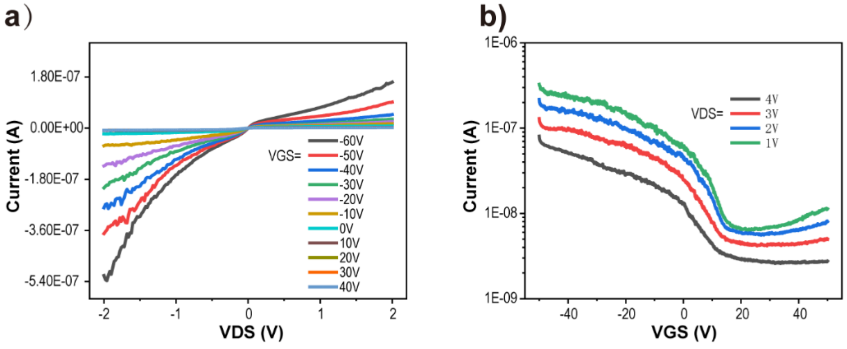

Figure S6. Silicon channel output (**a**) and transfer curves (**b**).

G. Growth of ZnO Nanowires by Hydrothermal Methods

For the growth of ZnO nanowires, the substrate was placed into 800 mL of a 40 mM solution of zinc nitrate hexahydrate and an equal amount of hexamethylenetetramine to increase ZnO for 1 hour (Figure S6b). Two 1000 mL bottles of 80 mM zinc nitrate hexahydrate and equal concentrations of hexamethylenetetramine solution were pumped into the reaction vessel after one hour using the peristaltic pump (Figure S6a). One peristaltic pump was used to pump in the solution while another pumped out the solution in order to maintain a stable volume of solution in the reaction vessel. A transparent silicone hose with an outer diameter of 3 mm and an inner diameter of 1 mm was used. A 1000 ml crystallization dish was used as the reaction vessel, and an acrylic plate was used as a table for placing the substrates. Zinc nitrate and hexamethylenetetramine solutions were introduced simultaneously into the vessel by means of a peristaltic pump operating at 10 revolutions per minute; the reaction solution was withdrawn from the container using a peristaltic pump operating at 20 rotations per minute. The reaction vessel was kept at a constant temperature of 85°C throughout the whole growth process, which lasted about 24 hours.

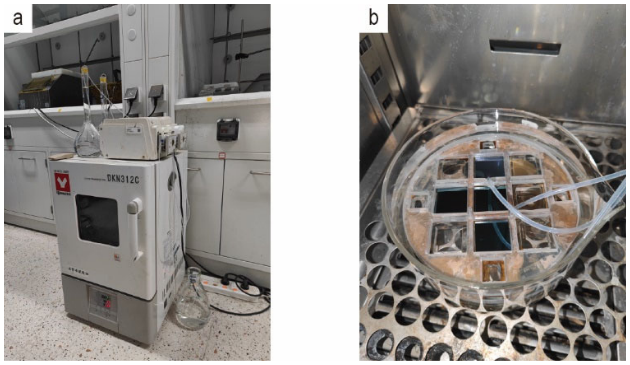

Figure S7. Growth of ZnO nanowires by hydrothermal means. Growth of ZnO nanowires using peristaltic pumps (**a**) and placement of the substrate on acrylic support (**b**).
